# Commentary: Coercion in Psychiatry: Lessons Learned from Trauma-Informed Care

**DOI:** 10.1177/07067437221125884

**Published:** 2022-09-21

**Authors:** Jonathan Jin, Lisa Burback, Andrew J. Greenshaw, Olga Winkler

**Affiliations:** 1 Department of Psychiatry, 3158 University of Alberta, Edmonton, Canada

**Keywords:** coercion, trauma-informed care, equity, restraints

## Coercion in Mental Health Settings

Coercion is the use of force or threat to compel another to act in a particular way. In the typical limited resources domain of psychiatry, with behavioural crises and reduced capacity to make treatment decisions, coercion is sometimes the only practical choice available to mitigate imminent harm. Historically, coercive practices in psychiatry are associated with iatrogenic harm, decreased patient satisfaction, and increased risk of suicide attempts, prompting international calls to reduce such practices. However, simple policies often cannot address the complexities of managing behavioural crises equitably to mitigate harm, so an alternative approach is needed. In psychiatric practice in Canada specifically, there is a lack of national strategy regarding patient safety.^1^ Trauma-informed care principles offer potential to reduce harmful coercive behaviours in alignment with equity, diversity, and inclusion (EDI) in medicine. Potential benefits of implementing trauma-informed care practice in psychiatry range from reduction of patient behaviours that may result in use of coercive practices, to addressing clinician biases. We believe these principles can help guide us to create national standards around patient safety in response to Waddell and Gratzer's^1^ call to action.

## Coercion by Restraints

A systematic review reported that amplifying the voices of patients and supporting staff help to mitigate harm from restraints and seclusion.^2^ While physical and chemical restraints are typically used non-consensually, variables that affect how a patient perceives restraints include feeling a “loss of self-respect and dignity” and “feeling less safe.” Debriefings with the patient and attempting to ensure that individuals feel heard while administering restraints reduces potential for psychological harm.^2^

## Coercion by Legal Orders Ensuring Adherence to Treatment

Three ways to legally mandate treatment in Canada include inpatient or community-based treatment orders, and certification under a mental health act. Community Treatment Orders (CTOs) are legal orders for a patient to adhere to supervised mental health treatment while living in the community. A systematic review of qualitative studies found patient experiences toward CTOs can be negative.^3^ For example, some participants reported a sense that they were “emotionally threatened,” had their choice taken away, and were being kept in a system to control them. Collaborative decision making throughout the treatment process reduces the negative impact of CTOs.^3^

## Trauma-Informed Care Reduces Iatrogenic Harm and Reliance on Coercion

The trauma-informed care approach seeks to recognize and understand how individuals’ past experiences inform their current presentation, so providers may respond with attention to individuals’ sense of psychological safety. This includes attending to uneven power dynamics within healthcare interactions, which may activate trauma responses to previous threatening experiences. This approach focuses on transparency, and a mindful, collaborative stance that maximizes patient empowerment and choice, and may be useful widely, including for those who experienced relational stress, attachment injury, childhood adversity, or were subject to cultural, gender, or racial stereotypes and injustices.^4^

A systematic review demonstrated that trauma-informed care implementation in inpatient settings is associated with reductions in seclusion and restraint use and in patient and staff injuries.^2^ One mixed-methods study found changes in nurses’ behaviour following trauma-informed care training, as one nurse articulated, “The person may have still needed to be restrained but we were able to use less restraint and I think a lot of that was through actually engaging the person in the whole process…so we were trying to work with them as much as possible as well.”^5^ In essence, the practice of trauma-informed care has the potential to reduce reliance on coercive practices, and in cases where coercion is the only choice, it can provide a buffer to the iatrogenic psychological harm that these practices can generate.

Competence in trauma-informed care requires acquisition of knowledge and the active practice of following its principles.^4^ For example, consider a situation of an Aboriginal man with agitation raising his voice in an emergency room. Instead of responding immediately with force or reiteration of hospital rules, trauma-informed care would imply that the clinician first recognizes the possibility that past adverse experiences might be playing a role in the situation, including potential cultural and historical trauma. First impressions, stereotypes or labels would be noticed but not acted upon. Instead, the agitation would be viewed as potentially a response to threat, and strategies to increase sense of safety would take precedence. These might include validation strategies, attending to clinician voice and body language, and asking the person what is needed to feel more at ease. This could take the form of incorporating knowledge about Aboriginal culture, engaging Aboriginal liaisons or simply demonstrating openness to understanding the person's experience. Shifting from a place of “knowing” to “not knowing” allows a clinician to assess this patient's unique history and how it influences current well-being, as well as any need for cultural or other facilitators. The practice of trauma-informed care endeavours to create a socially safe environment for both parties to treat the other with respect and dignity, with empathy and compassion often naturally following. In cases of moral dilemmas where coercion is deemed necessary to prevent physical harm, trauma-informed care provides direction for service providers to exercise caution and reduce psychological harm by maximizing transparency and collaborative decision making wherever possible.^5^ For example, this might include offering options, such as oral or injectable medications or restraints, framing them as temporary methods to ensure physical safety and help the person regain control over his or her behaviours, and indicating a willingness to collaborate about how this will be monitored and reassessed, leveraging a recovery framework,^6^ and to reduce a sense of helplessness or lack of control.

## Relationship with EDI

In addition to reducing coercion, a shift to trauma-informed care will likely facilitate EDI for broader systems, provided that leadership is trauma-informed. At its core, EDI prioritizes removal of systemic barriers to fair treatment and access, having a respect for individual differences, and ensuring that all voices are valued—such priorities are naturally addressed when trauma-informed care is implemented. The literature detailing efforts to incorporate EDI in healthcare is nascent but rapidly emerging, as EDI, along with an intersectional lens, is embraced as a desired set of values.^7^ Trauma-informed care assumes that everyone has a unique perspective, informed by past experiences and cultural and historical context, while avoiding stereotypes, lending to respect for diversity. Recognizing the impact of historical and cultural aspects to adversity and traumatic experience can increase sensitivity to factors impacting equity. By reducing assumptions, increasing empathy, and valuing transparency, safety and collaborative decision making, trauma-informed care can facilitate improved connection with others and increased sense of inclusivity.

The need to implement trauma-informed care in psychiatry is highlighted by recent epidemiological research supporting the significant role of past adversity and trauma in mental illness across a range of psychiatric diagnoses, and the high prevalence of trauma reported in marginalized groups such as ethnic and gender minorities.^8^ Although not all patients will develop mental health sequelae from this exposure, trauma history has been shown to negatively impact wellness and be associated with socioeconomic disadvantage.^8^ Whether addressing overt traumatic sequelae or the deleterious effects of microaggressions and discrimination,^9^ trauma-informed care may offer a concrete step forward in training clinicians to be aligned with EDI priorities in clinical practice and improve patient safety in Canadian psychiatric practice, in keeping with Waddell and Gratzer's^1^ call to action.
